# Immunogenicity evaluation of recombinant *Lactobacillus casei* W56 expressing bovine viral diarrhea virus E2 protein in conjunction with cholera toxin B subunit as an adjuvant

**DOI:** 10.1186/s12934-020-01449-3

**Published:** 2020-10-01

**Authors:** Shuo Jia, Xinning Huang, Hua Li, Dianzhong Zheng, Li Wang, Xinyuan Qiao, Yanping Jiang, Wen Cui, Lijie Tang, Yijing Li, Yigang Xu

**Affiliations:** 1 Heilongjiang Key Laboratory for Animal Disease Control and Pharmaceutical Development, College of Veterinary Medicine, Northeast Agricultural University, Harbin, P. R. China; 2 Northeast Science Inspection Station, Key Laboratory of Animal Pathogen Biology of Ministry of Agriculture of China, Harbin, P. R. China

**Keywords:** Bovine viral diarrhea virus (BVDV), E2 protein, Cholera toxin B subunit (ctxB), Recombinant *lactobacillus* vaccine, Immunogenicity

## Abstract

**Background:**

Bovine viral diarrhea virus (BVDV) is one of the main causes of infectious diseases in cattle and causes large financial losses to the cattle industry worldwide. In this study, *Lactobacillus casei* strain W56 (Lc W56) was used as antigen deliver carrier to construct a recombinant *Lactobacillus* vaccine pPG-E2-ctxB/Lc W56 constitutively expressing BVDV E2 protein fused with cholera toxin B subunit (ctxB) as an adjuvant, and its immunogenicity against BVDV infection in mice model by oral route was explored.

**Results:**

Our results suggested that pPG-E2-ctxB/Lc W56 can effectively activate dendritic cells (DCs) in the Peyer’s patches, up-regulate the expression of Bcl-6, and promote T-follicular helper (Tfh) cells differentiation, as well as enhance B lymphocyte proliferation and promote them differentiate into specific IgA-secreting plasma cells, secreting anti-E2 mucosal sIgA antibody with BVDV-neutralizing activity. Moreover, significant levels (*p* < 0.01) of BVDV-neutralizing antigen-specific serum antibodies were induced in the pPG-E2-ctxB/LC W56 group post-vaccination. The recombinant *Lactobacillus* vaccine can induce cellular immune responses, and significant levels (*p* < 0.01) of Th1-associated cytokines (IL-2, IL-12, and IFN-γ), Th2-associated cytokines (IL-4, IL-10) and Th17-associated cytokine (IL-17) were determined in the serum of vaccinated mice. Significantly, the recombinant *Lactobacillus* vaccine provides immune protection against BVDV infection, which can be cleared effectively by the vaccine post-challenge in orally vaccinated animals.

**Conclusions:**

The genetically engineered *Lactobacillus* vaccine constructed in this study is immunogenic in mice and can induce mucosal, humoral, and cellular immune responses, providing effective anti-BVDV immune protection. It thus represents a promising strategy for vaccine development against BVDV.

## Background

Bovine viral diarrhea virus (BVDV) is the causative pathogen of bovine viral diarrhea-mucosal disease (BVD-MD), resulting in significant economic losses to the cattle industry worldwide [1, 2]. BVDV, belonging to the genus *Pestivirus*, the family *Flaviviridae*, is an enveloped single-stranded RNA virus. According to the differences of genetic and antigenic, BVDV is divided into two genotypes, type 1 (BVDV-1) and type 2 (BVDV-2). Further, according to their effects on cultured cells, each genotype includes two biotypes, cytopathic (CP) and noncytopathic (NCP) [3, 4]. BVDV infection in cattle can cause various clinical symptoms, such as respiratory diseases, mucosal diseases, immunosuppression, diarrhea, and persistent infection (PI) [5, 6]. BVDV can also infect other mammals, including sheep, goats, deer, camels, and pigs [7–9]. There are currently several inactivated vaccines and live attenuated BVDV vaccines available commercially. Although both types of traditional vaccines play an important role in controlling BVDV infection, both have their risks and drawbacks [1, 10–12]. In the case of inactivated or attenuated BVDV vaccine, their effects are controversial in practice under controlled experimental conditions and field conditions [13]. This is particularly true for attenuated CP-BVDV vaccines, which can induce serious mucosal disease in PI calves. Therefore, the design of new vaccine strategies is necessary in order to prevent future BVDV infections.

Generally, BVDV infection often initiates at the oronasal mucosa and intestinal mucosa [14, 15]. Thus, the synergistic effect of the local mucosal immune response and systemic immune response is important to inhibit BVDV from invading the body through the mucosa tissue and then spreading to the systemic circulation. An oral mucosal vaccine is a promising vaccine strategy against BVDV infection as it could effectively induce secretory IgA (sIgA)-based mucosal immune response and IgG-based systemic immune response that target specific immunogens, thus preventing the virus from invading, replicating at mucosal tissue, and further spreading to other tissues. For this purpose, effective antigen delivery carrier is needed in order to deliver the antigen in an immunogenic and protected form to the targeted mucosa, where it will trigger mucosal and systemic immune responses post oral vaccination. The use of lactic acid bacteria as antigen delivery carriers represents a promising approach to express heterologous antigens for the development of oral vaccines, which has attracted much attention in this field [16–19], particularly in terms of the use of *Lactobacillus* strains [20–25], which can be used as a safe carrier with a good tolerance to gastrointestinal conditions and the ability to colonize the intestinal tract.

E2 protein, the major envelope glycoprotein of BVDV, has important epitopes that elicit neutralizing antibodies to protect against infection, and is important for the virus attach to host cells, which can help the virus to enter into the cells, where it completes its replication and proliferation [26]. Importantly, the E2 protein encompasses the major BVDV-neutralizing antigenic sites, and can induce the production of BVDV-neutralizing antibody, which is important for preventing BVDV infection [27–30], suggesting a prospective candidate that can be used as an immunogen from which to construct a vaccine against BVDV. In addition, the combination of antigens with vaccine adjuvants, comprised of components for the induction of potent immune responses, can effectively increase the titers of antibody, induce immune responses rapidly, and reduce the requirement of the dose of antigen [31]. As an adjuvant, cholera toxin B subunit (ctxB) contributes to promote the absorption of proteins in the intestinal tract and significantly enhance antigen-specific immune responses at mucosal surfaces [32]. Moreover, ctxB can also be used as an adjuvant to increase the permeability of intestinal mucosal cells and reduce the antigen dose used for vaccination, due to its ability to bind efficiently to antigen-presenting cells, thus further accelerating protective immune responses against infections [33, 34], indicating its high potential as an immunological adjuvant and immunomodulator.

In conclusion, a genetically engineered *Lactobacillus* vaccine pPG-E2-ctxB/Lc W56 constitutively expressing BVDV E2 protein in conjunction with ctxB as an adjuvant was constructed using *Lactobacillus casei* strain W56 that was isolated from cattle feces [23] as an antigen delivery carrier. The immunogenicity and protective efficacy of the recombinant *Lactobacillus* vaccine against BVDV infection was evaluated after oral vaccination.

## Methods

### Bacteria, virus, plasmids, primers, and mice

*Lactobacillus casei* strain W56 (Lc W56) was isolated from cattle feces followed by identification of morphology, biochemical reaction, and 16S rDNA sequencing in our laboratory and was cultured in de Man, Rogosa, and Sharpe (MRS) broth (Sigma, St.Louis, MO, USA) at 37 °C. The strain Lc W56 shows good probiotics proprieties, including good colonization ability in intestine [23], tolerance to extreme digestive environments (hypertonic environment of 9% NaCl, 0.5% bile, and gastric environment of pH 1.5) and intestinal fluid environment. Moreover, the strain Lc W56 can effectively promote animal growth performance and protect animal against enteropathogenic bacteria (*Salmonella typhimurium* and *Escherichia coli* K99) infection. *Vibrio cholerae* strain OG80 was kept in our laboratory. Cytopathic (CP) BVDV-1 strain ZD-2018 was propagated in MDBK cells at 37 °C with 5% CO_2_, which was isolated from the calf suffering from diarrhea by our laboratory and identified by virus propagation, indirect immunofluorescence assay, and gene sequencing. The constitutive expression plasmid pPG-T7g10-PPT was constructed in our lab. The recombinant plasmid pMD-E2 containing the full-length E2 gene of BVDV strain ZD-2018 was constructed in our lab [23]. Five-week-old specific pathogen-free (SPF) BALB/c mice were obtained from Changsheng Biotechnology Company (Shenyang, China), and feed by a standard mouse diet and water. Primers were designed in this work by the Oligo 6.0 software, and details of primers were provided in Table 1.

**Table 1:** Details of primers used in this study

Primers	Sequences (5′–3′)
E2-F	GAGCTC^a^ATGCTCCCAGCCTGTAAACC
E2-R	GGGCCCAAACCGGAATTCACCTAAGGTCGTTTGTTCTGAT
ctxB-F	GAATTCGGTGGTGGTGGTTCTGGTGGTGGTGGTTCT^b^ATGATTAAATTAAAATTTGGTGTTT
ctxB-R	GGGCCCTTAATTTGCCATACTAATTGC
5′UTR-F	GGTAGCAACAGTGGTGAG
5′UTR-R	GTAGCAATACAGTGGGCC

^a^Underlining indicates restriction enzyme recognize site

^b^Italic indicates the gene encoding flexible linker (GGGGS)2

### Construction of genetically engineered *Lactobacillus* pPG-E2-ctxB/Lc W56

**Fig. 1:**
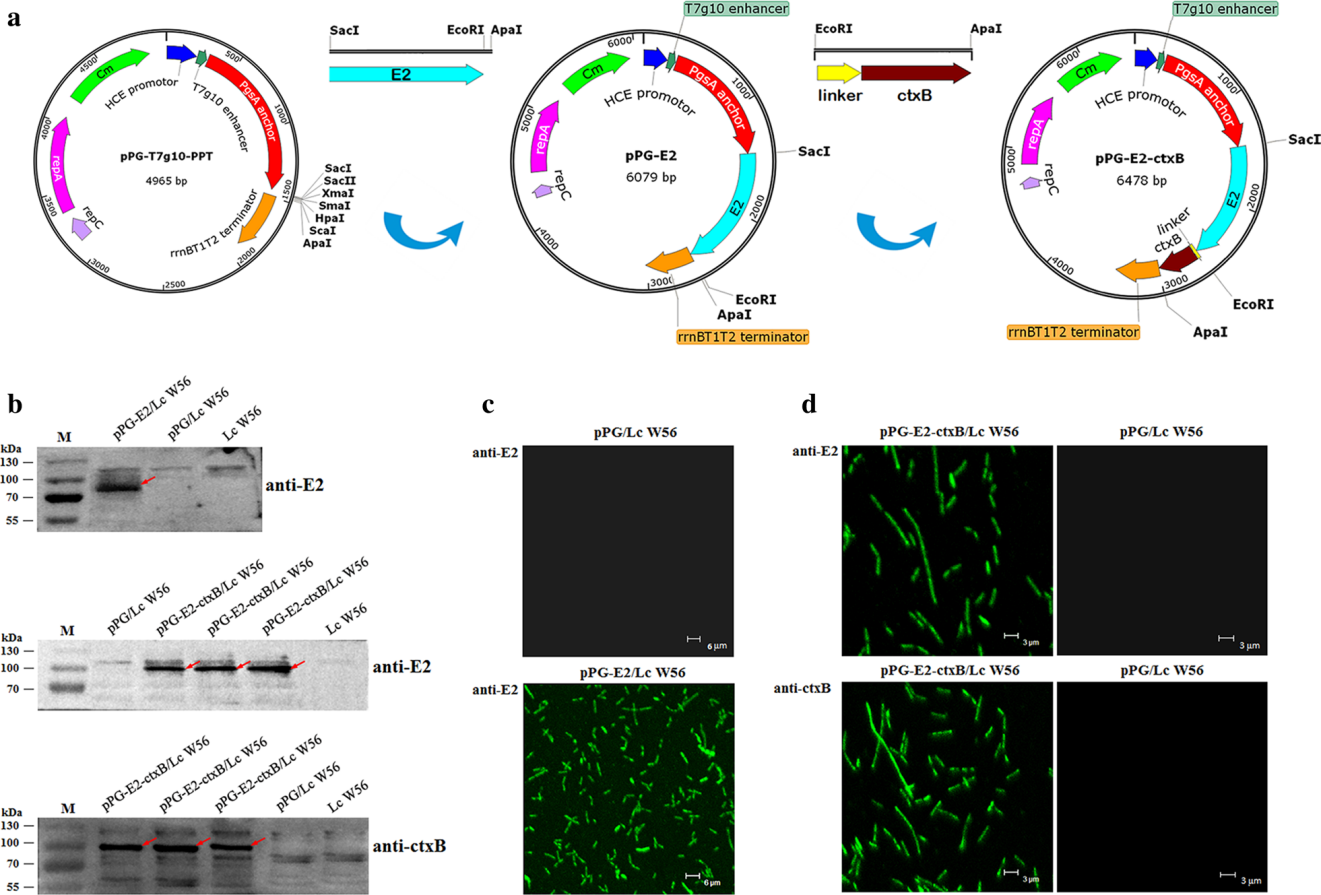
Construction of the recombinant *Lactobacillus* strains and identification of the proteins of interest expressed by the recombinant *Lactobacillus* strains. **a** Schematic illustration of the construction of the recombinant pPG-E2 and pPG-E2-ctxB plasmids. **b** Identification of the proteins of interest expressed by the recombinant strains pPG-E2/Lc W56 and pPG-E2-ctxB/Lc W56 using Western blot with mouse anti-E2 or anti-ctxB mAb, respectively. **c** Identification of the E2 protein expressed by pPG-E2/Lc W56 by IFA. **d** Identification of the fusion protein E2-ctxB expressed by pPG-E2-ctxB/Lc W56 by IFA

The process of constructing recombinant *Lactobacillus* pPG-E2-ctxB/Lc W56 is illustrated in Fig. 1a. Briefly, we used the pMD-E2 plasmid as a template to amplify the full-length E2 gene by PCR with the primer pair E2-F/E2-R containing *Sac* I site and *Apa* I restriction site, respectively. This PCR product was then subcloned as a *Sac* I and *Apa* I gene fragment into the plasmid pPG-T7g10-PPT, generating the recombinant plasmid pPG-E2. Subsequently, the whole gene encoding ctxB was obtained by PCR amplification with the primer pair ctxB-F/ctxB-R, using the genomic DNA of *V*. *cholerae* OG80 as a template that was extracted by a Bacterial Genomic DNA Extraction kit (Thermo Fisher Scientific, San Jose, CA, USA). This PCR product was then digested with EcoRI and ApaI and inserted into the plasmid pPG-E2 to obtain the recombinant plasmid pPG-E2-ctxB, of which E2 and ctxB were linked by a flexible linker (GGGGS)2. Next, the plasmid pPG-E2-ctxB was electroporated into the Lc W56 competent cells, generating the recombinant *Lactobacillus* pPG-E2-ctxB/Lc W56. The preparation of competent cells and electroporation were performed as the method described previously [35–37]. Briefly, Lc W56 was cultured in MRS broth until OD_600_≈0.3 for preparing competent cells; then, 50 ng of plasmid pPG-E2-ctxB was gently mixed with 200 µL of Lc W56 competent cells, transferred into a pre-cooled Gene Pulser™ cuvette (inter-electrode distance of 0.2 cm), and placed into a Gene Pulser (Bio-Rad, Hercules, CA, USA) followed by a single electric pulse (2,500 V/cm; 25 µF); the treated Lc W56 was grown in recovery medium (MRS broth supplemented with 0.3 M sucrose), and the positive recombinant strain was selected on MRS agar medium containing 10 μg/mL chloromycetin.

### Identification of the fusion protein of interest

Recombinant *Lactobacillus* pPG-E2-ctxB/Lc W56 was grown in MRS broth at 37 °C for 16 h. After centrifugation at 10,000×*g* for 5 min, harvested the bacteria and resuspended with 2× sodium dodecyl sulfate (SDS) buffer, then placed the cells in boiling water for 10 min followed by another round of centrifugation. Subsequently, the proteins in the supernatants were separated using 12% SDS–polyacrylamide gel electrophoresis (SDS-PAGE), followed transferred onto a polyvinylidene fluoride (PVDF) membrane. Mouse anti-E2 (1:500) or anti-ctxB (1:200) monoclonal antibody (mAb) prepared by our laboratory were used as the primary antibody, and the horseradish peroxidase (HRP)-conjugated goat anti-mouse IgG antibody (1:2000) (Sigma, USA) were used as the secondary antibody, respectively. Immunoblotting were taken by a chemiluminescent substrate reagent (Thermo Fisher Scientific, USA). In addition, an indirect immunofluorescence assay (IFA) was performed to detect the expression of the fusion protein in the recombinant *Lactobacillus* pPG-E2-ctxB/Lc W56. In brief, the pPG-E2-ctxB/Lc W56 grown overnight in MRS broth was washed two times with sterile PBS followed by centrifugation at 10,000×*g* for 10 min. Next, the pellets were incubated with mouse anti-E2 mAb (1:500) or anti-ctxB mAb (1:200) as the primary antibody, and fluorescein isothiocyanate (FITC)-labeled goat anti-mouse IgG antibody (1:1000) (Thermo Fisher Scientific, USA) as the secondary antibody. After washing three times with PBS, the fluorescence signals on the cell surface of recombinant strain were observed by laser confocal microscopy. Moreover, the expression levels of E2 protein in the pPG-E2-ctxB/Lc W56 and pPG-E2/Lc W56 were analyzed by Western blot assay. Briefly, one milliliter of the recombinant strain pPG-E2-ctxB/Lc W56 and pPG-E2/Lc W56 (OD_600_ ≈ 1.0) were centrifugated and lysed, respectively; after adjusting total protein concentration of each recombinant strain sample to be the same, Western blot assay was carried out with the mouse anti-E2 mAb to analyze the level of E2 protein expressed in the pPG-E2-ctxB/Lc W56 and the pPG-E2/Lc W56.

### Tolerance of the pPG-E2-ctxB/Lc W56 to digestive environments

The experiments were performed according to the methods described previously [23]. Briefly, to test the tolerance to hypertonic environment and bile, the recombinant strain pPG-E2-ctxB/Lc W56 of OD_600_ ≈ 1.0 was centrifuged, reinoculated respectively in 1 mL of MRS broth supplemented with 4–9% of NaCl, and 0.05–0.5% of bile (wt/vol), and continually cultured at 37 °C for 5 h followed by counting live bacteria using plate method. To test the tolerance to intestinal fluid environment, the recombinant strain pPG-E2-ctxB/Lc W56 (OD_600_ ≈ 1.0) was reinoculated in 1 mL of simulated intestinal fluid at 37 °C for 3 h followed by counting live bacteria using plate method at 30-min intervals. Significantly, we tested the tolerance of the pPG-E2-ctxB/Lc W56 to rumen fluid of cattle in vitro. The recombinant strain of OD_600_≈1.0 was reinoculated in 1 mL of rumen fluid of cattle with pH 1.5, 2.5, 3.5, or 4.5, and continually cultured at 37 °C for 2.5 h followed by counting live bacteria using plate method.

### Activation of dendritic cells in Peyer’s patches by the recombinant *Lactobacillus* vaccine

On day 7 after oral vaccination, the Peyer’s patches (PPs) in the small intestine of mice (*n* = 5) in the pPG-E2-ctxB/Lc W56 group were isolated to detect the activation of dendritic cells (DCs) induced by the recombinant strain pPG-E2-ctxB/Lc W56. Briefly, PPs were harvested from the small intestine of the mice in each group, placed in 5 mL of pre-cooled Hank’s Balanced Salt Solution (HBSS), grinded gently, and filtered using a 200-mesh stainless steel mesh, followed by centrifugation at 500×*g* for 10 min. Subsequently, the cells were then resuspended using 8 mL HBSS, placed into 5 mL of 70% Percoll (Sigma, USA) solution in 15 mL centrifuge tubes, followed by centrifugation at 500×*g* for 20 min. The cells on the interface were collected and washed two times with HBSS, then resuspended by HBSS at a concentration of 10^6^/mL. After that, the harvested cells were incubated with anti-CD16/CD32 antibody (Abcam, Cambridge, MA, USA) to block the Fc receptors, washed two times with HBSS, and stained with FITC-conjugated anti-CD40 and CD86 antibodies (Abcam, Cambridge, MA, USA). Finally, the samples were analyzed by flow cytometry. As a control, cells were collected from the pPG-E2/Lc W56, pPG/Lc W56, and PBS groups. Each experiment was performed in triplicate.

### Effects of the recombinant *Lactobacillus* on differentiation of IgA-secreting cells in PPs

On day 7 after oral vaccination with the recombinant strains, the expression of Bcl-6 in T lymphocytes of the PPs obtained from the mice (*n* = 5) in pPG-E2-ctxB/Lc W56, pPG-E2/Lc W56, pPG/Lc W56, and PBS groups was analyzed using an immunohistochemistry (IHC) assay. Briefly, the PPs were isolated from the mice in these groups, and then fixed with 10% formaldehyde solution, coated with paraffin, followed by the establishment of tissue sections. Next, the tissue sections were incubated with mouse anti-Bcl-6 mAb (Abcam, MA, USA) at 37 °C for 2 h, followed by incubation with HRP-conjugated goat anti-mouse IgG (Sigma, USA) and FITC-conjugated goat anti-mouse IgG (Thermo Fisher Scientific, CA, USA), respectively. Subsequently, the expression of Bcl-6 was visually evaluated using an inverted microscope. At the same time, the PPs isolated from the mice (*n* = 5) in these groups were grinded gently using a 300-mesh stainless steel mesh and rinsed with PBS. After centrifugation at 1000×*g* for 5 min, the resulting cells were resuspended in 0.5 mL PBS, followed by staining with the corresponding antibodies (2 µl of anti-mouse CXCR5/APC and anti-mouse CD4/PE, respectively). The percentage of CD4^+^CXCR5^+^ T cells were detected by flow cytometry (cells without staining were used as negative control, and cells stained with CXCR5/APC or CD4/PE were used as compensation controls, and the cells labeled with both CXCR5 and CD4 are regarded as CD4^+^CXCR5^+^ T cells). In addition, the collected cells were incubated by anti-mouse IgA/FITC, anti-mouse B220/PerCP-Cy5, and anti-mouse IgM/PE-Cy7, and the percentage of B220^+^IgM^+^ B lymphocytes, B220^+^IgA^+^ B lymphocytes, and B220^−^IgA^+^ plasma blast was then determined by flow cytometry. The experiment was performed in parallel triplicate repeats.

### Immunization

The recombinant strain pPG-E2-ctxB/Lc W56 cultured for 12 h in MRS broth was washed once and resuspended with PBS containing 5% casein peptone and 0.5% glucose at the concentration of 10^10^ CFU/mL. The mice (*n* = 50) were orally vaccinated with 200 μL of recombinant pPG-E2-ctxB/Lc W56 by the gavage needles. The mice were vaccinated consecutively for three days as previously described [23]. In brief, on days 1–3, primary vaccination was given, then days 15–17, is time for the second vaccination and on days 28–30, the final vaccination was given. In parallel, the mice in other three groups (50 mice per group) that were orally administered with pPG-E2/Lc W56, pPG/Lc W56, and PBS were used as control. The samples including serum, feces, intestinal mucus, nasal fluid, and genital tract fluid were harvested from mice (*n* = 5) in each group on days 0, 7, 14, 21, 28, 35, 42, 49, 56, and 63 after primary vaccination. All samples were treated as methods previously described [22, 23], and then stored at − 80 °C until used.

### ELISA and IHC assay for antigen-specific IgA-secreting cells in PPs

The levels of anti-BVDV IgG antibody in the serum and anti-BVDV sIgA antibody in the intestinal mucus, nasal fluid, genital tract fluid, and feces samples were detected by ELISA. In brief, 96-well polystyrene plates were coated with 200 TCID_50_ (10^7.2^/0.1 mL) BVDV strain ZD-2018 at 4 °C for 12 h. After washing three times with PBST (PBS containing 0.1% Tween-20), the plates were blocked with 5% skim milk at 37 °C for 2 h, followed by washing with PBST. Next, the serum sample (1:100) and other samples (1:10) used as the primary antibodies were added into the plates and kept in 37 °C for 1 h, followed by washing three times with PBST. Subsequently, HRP-conjugated goat anti-mouse IgG or IgA antibody (1:5000) (Sigma, USA) was added as the secondary antibody into the plates, and kept in 37 °C for 1 h. After washing three times with PBST, the substrate o-phenylenediamine dihydrochloride was used for color development, followed by the measurement of absorbance at 450 nm. On the 42d post primary vaccination, the levels of cytokines IL-2, IL-4, IL-10, IL-12, IL-17, and IFN-γ in the serum of these groups were analyzed by ELISA Kits (Abcam, Cambridge, MA, USA). Moreover, an IHC assay was performed, as described above, to detect the antigen-specific sIgA-secreting cells in the PPs of the mice collected from each group using the anti-E2 sIgA antibody induced by pPG-E2-ctxB/Lc W56.

### Determination of BVDV-neutralizing ability of antibodies

On the 42 days post primary vaccination, the neutralizing ability against BVDV of serum IgG and mucosal sIgA antibodies collected from four groups was determined. Briefly, 100 µL of antibody (serum IgG and mucosal sIgA) samples were diluted serially (twofold) in a 96-well plate, followed by the addition of equal volumes of BVDV strain ZD-2018 (200 TCID_50_) into the plate and incubation at 37 °C for 1 h. The antibody-BVDV mixture were then transferred onto a MDBK cell monolayer and incubated at 37 °C with 5% CO_2_ for 3 days, after which we evaluated the cytopathic effects (CPE). Five technical (five samples) with eight biological replicas for each sample were taken for each group. The neutralizing ability of each sample was calculated using the Reed-Muench method and the result of each group was shown as mean ± standard deviation (SD).

### Lymphoproliferation activity test

On the 42 days post the primary vaccination, the splenocytes of mice (*n* = 5) in these four groups were collected, adjusted to a final concentration of 5 × 10^6^ cells/mL in RPMI 1640 with 10% fetal bovine serum (FBS) (Sigma, USA), and then added cells into a 96-well plate with eight duplicates (100 μL/well) followed by incubation at 37 °C in 5% CO_2_ for 12 h. The cells were then stimulated using recombinant E2 protein at a concentration of 1, 5, and 25 µg/mL for three days, respectively. In parallel, sample stimulated with 5 µg/mL of concanavalin A, ConA (Sigma, USA) was used as the positive control and RPMI 1640 was used as a mock stimulation control. After adding 10 μL of thiazolyl blue tetrazolium bromide (MTT) (Sigma, USA) at a concentration of 5 mg/mL into each well, the plate was kept in 37 °C for 4 h, and the absorbance of each well was determined at 570 nm. The stimulation index (SI) was calculated as follows: SI = OD_570_(sample)/OD_570_(control). In addition, the T lymphocytes subsets in the splenocytes of mice in different groups were detected by flow cytometry after oral vaccination. Briefly, splenocytes with the concentration of 5 × 10^6^ cells/mL harvested from these four groups on the 42d post primary vaccination were incubated with anti-mouse CD4/PE antibody and anti-mouse CD8/APC antibody (Abcam, USA) at 37 °C for half an hour, followed by flow cytometry analysis.

### Challenge

A challenge experiment was carried out to determine the protective effect of the recombinant *Lactobacillus* vaccine after oral vaccination using mice as the animal model. In brief, a total of 240 mice were separated into four groups (60 mice per group) and were orally vaccinated with pPG-E2-ctxB/Lc W56, pPG-E2/Lc W56, pPG/Lc W56, and PBS, respectively, according to the vaccination protocol described above. On the 42d post primary vaccination, each mouse in four groups was challenged with 200 µL of 10^5^ TCID_50_ BVDV strain ZD-2018 propagated in MDBK cells. During a 12d challenge period, the intestine, lung, spleen, blood, and feces samples were collected from the mice (*n* = 5) in each group per day, and the viral loads in the intestine, lung, spleen, and blood samples were detected using quantitative RT-PCR (RT-qPCR) with the primer pair 5′UTR-F/5′UTR-R. The viral load of the feces sample was detected using RT-PCR with the same primer pair, 5′UTR-F/5′UTR-R. On day 12 post BVDV infection, the histopathological changes in the lung, liver, kidney, heart, spleen, and small intestine of mice in each group were observed after hematoxylin–eosin (HE) staining. The viral antigens in the intestinal mucosa of the mice were detected using an IHC assay with HRP-conjugated mouse anti-E2 mAb prepared by our laboratory.

### Statistical analysis

In this work, results were shown as mean ± standard errors (SE) of three replicates each test in a single experiment repeated three times. Tukey’s multiple comparison tests and one-way analysis of variance (ANOVA) were used to analyze the differences among groups. * (*p* < 0.05), ** (*p* < 0.01), and *** (*p* < 0.001) was considered as significant, which was analyzed by GraphPad Prism V5.0 software.

## Results

### Identification of the fusion protein expressed by recombinant *Lactobacillus*

The protein of interest expressed by the recombinant *Lactobacillus* was identified by Western-blotting with mouse anti-E2 mAb and anti-ctxB mAb. We found that the E2 protein and the fusion protein (E2-ctxB) were constitutively expressed by the pPG-E2/Lc W56 and pPG-E2-ctxB/Lc W56, respectively (Fig. 1b). However, there was no specific immunoblotting band of the expected size observed for pPG/Lc W56 or Lc W56. Moreover, indirect IFA was also used to identify the expression of target proteins. Our results showed that specific levels of green fluorescence were observed on the surface of the recombinant *Lactobacillus* pPG-E2/Lc W56 and pPG-E2-ctxB/Lc W56, but not on the surface of pPG/Lc W56 and Lc W56 (Fig. 1c, d). In addition, the expression levels of E2 protein in pPG-E2-ctxB/Lc W56 and pPG-E2/Lc W56 were determined using Western-blotting assay, and there was no significant difference observed for pPG-E2-ctxB/Lc W56 and pPG-E2/Lc W56.

### Testing result of tolerance of pPG-E2-ctxB/Lc W56 to digestive environments

**Fig. 2:**
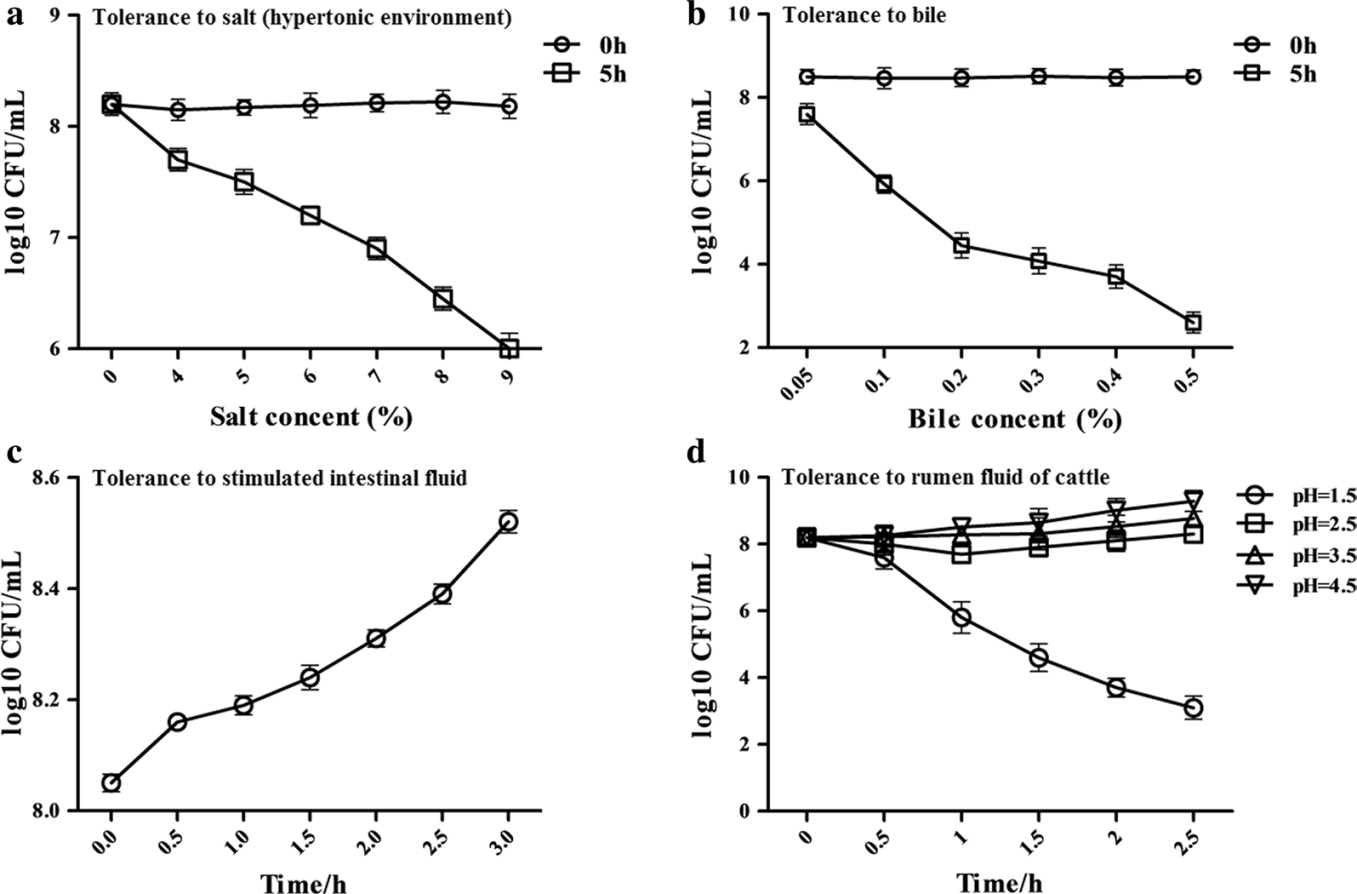
The tolerance of pPG-E2-ctxB/Lc W56 to different digestive environments. **a** Tolerance to salt (hypertonic environment). **b** Tolerance to bile. **c** Tolerance to simulated intestinal fluid environment. **d** Tolerance to cattle rumen fluid

In this work, we determined the tolerance of the recombinant pPG-E2-ctxB/Lc W56 to a hypertonic environment, bile, the intestinal fluid environment, and particularly the tolerance to the rumen fluid of cattle. Our results showed that when cultured in a hypertonic environment of 9% NaCl for 5 h (Fig. 2a), 0.5% bile for 5 h (Fig. 2b), intestinal fluid for 3 h (Fig. 2c), and rumen fluid of pH 1.5 for 2.5 h (Fig. 2d), pPG-E2-ctxB/Lc W56 maintained a high survival rate, indicating that the recombinant *Lactobacillus* pPG-E2-ctxB/Lc W56 has a certain level of tolerance to extreme digestive environments.

### Detection of DCs in PPs activated by the pPG-E2-ctxB/Lc W56

**Fig. 3:**
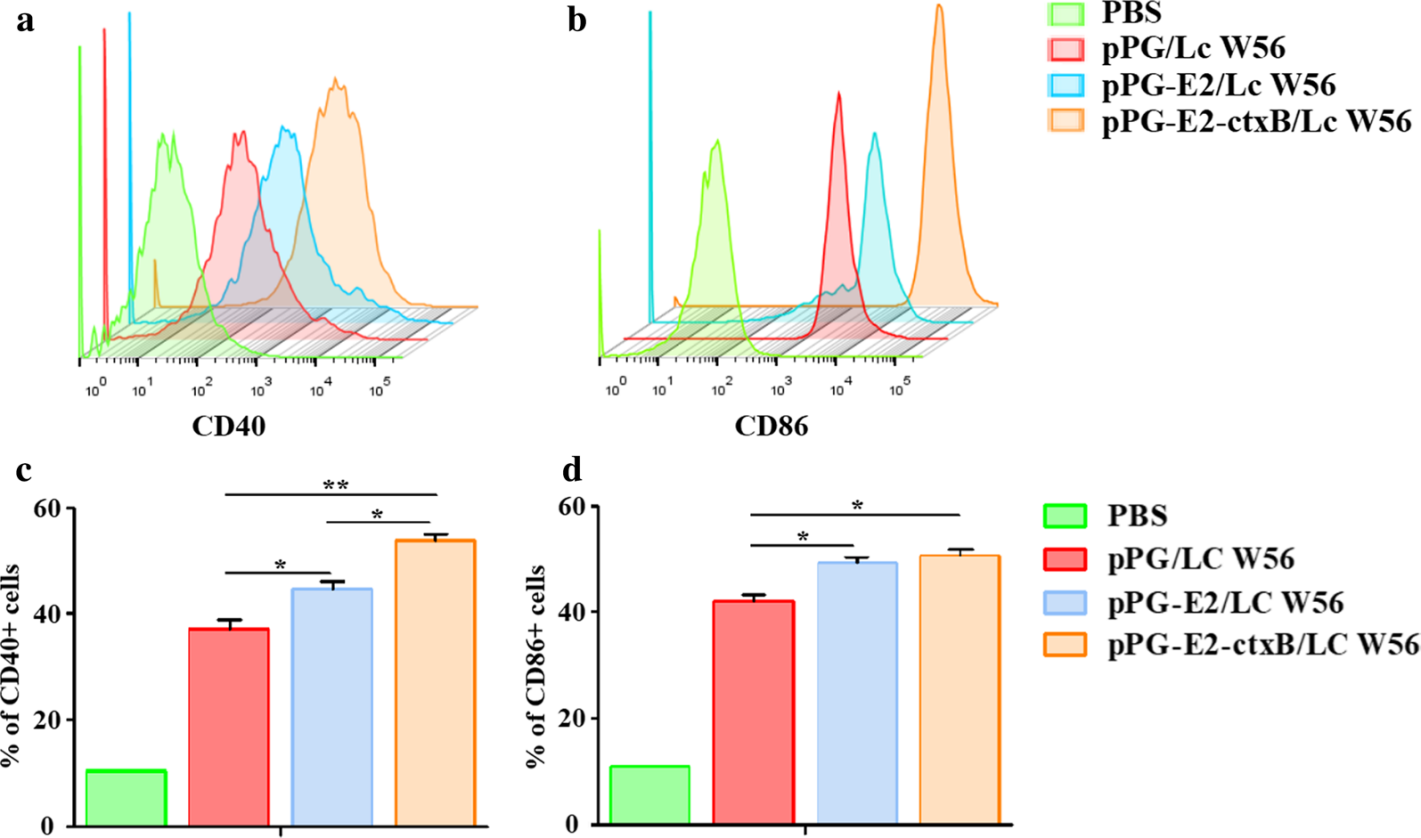
Activation of DCs in intestinal Peyer’s patches stimulated by the recombinant *Lactobacillus* strains pPG-E2/Lc W56 and pPG-E2-ctxB/Lc W56. On day 7 after oral vaccination, the cells in the intestinal PPs of the mice in each group were harvested and analyzed by flow cytometry with FITC-conjugated anti-CD40 antibody (**a**) and CD86 antibody (**b**) staining. The percentage of CD40^+^ and CD86^+^ cells of each group is shown in **c**, **d** respectively. Bars represent the mean ± SE of each group. * (*p* < 0.05), and ** (*p* < 0.01)

As shown in Fig. 3, the expression levels of CD40 (A) and CD86 (B) of DCs in the PPs isolated from the mice in each group were detected by flow cytometry on the 7d after oral vaccination. Compared to the PBS group, the expression levels of CD40 and CD86 of DCs stimulated by pPG-E2-ctxB/Lc W56, pPG-E2/Lc W56, and pPG/Lc W56 were all increased significantly (*p* < 0.001). The level of CD40 stimulated by pPG-E2-ctxB/Lc W56 was significantly higher than that stimulated by pPG-E2/Lc W56 (*p* < 0.05) and pPG/Lc W56 (*p* < 0.01). Moreover, the level of CD40 stimulated by pPG-E2/Lc W56 was significantly higher than that stimulated by pPG/Lc W56 (*p* < 0.05) (Fig. 3c), while the level of CD86 stimulated by pPG-E2-ctxB/Lc W56 and pPG-E2/Lc W56 was significantly higher than that stimulated by pPG/Lc W56 (*p* < 0.05). There was no significant difference in the level of CD86 stimulated by pPG-E2-ctxB/Lc W56 and pPG-E2/Lc W56 (Fig. 3d). Our data showed that pPG-E2-ctxB/Lc W56 and pPG-E2/Lc W56 could effectively promote the maturation of DCs. In addition, our results also indicated that Lc W56 had modulatory effect on dendritic cell function.

### Differentiation of IgA-secreting cells regulated by the pPG-E2-ctxB/Lc W56

**Fig. 4:**
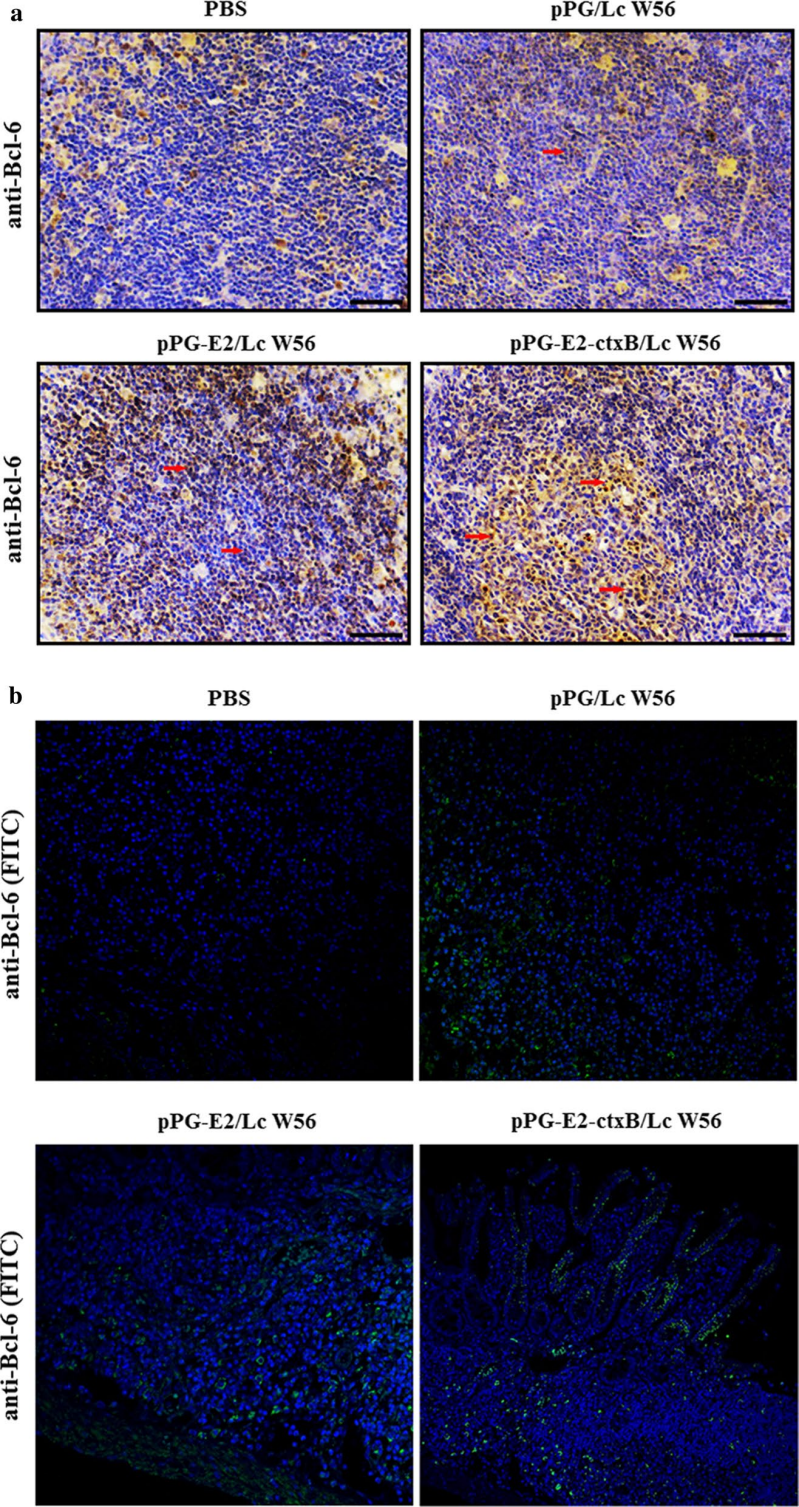
Detection of T lymphocytes expressing Bcl-6 in the PPs of the mice in each group on day 7 after oral vaccination by IHC assay (**a**) and fluorescence IHC assay (**b**)

**Fig. 5:**
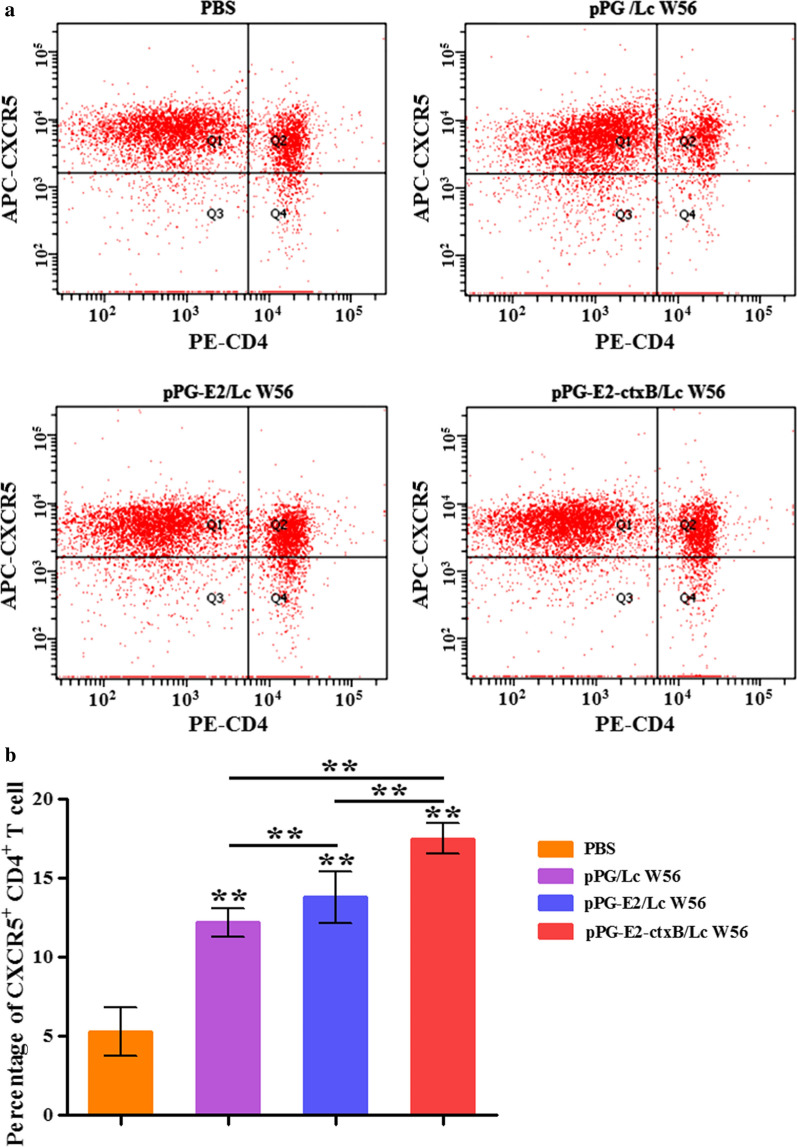
The level of CD4^+^CXCR5^+^ T cells in the intestinal Peyer’s patches of the mice in each group was determined by flow cytometry (**a**) on day 7 after oral vaccination. The percentage of CD4^+^CXCR5^+^ T cells of each group are shown in **b**. Bars represent the mean ± SE of each group. *(*p* < 0.05), and **(*p* < 0.01)

**Fig. 6:**
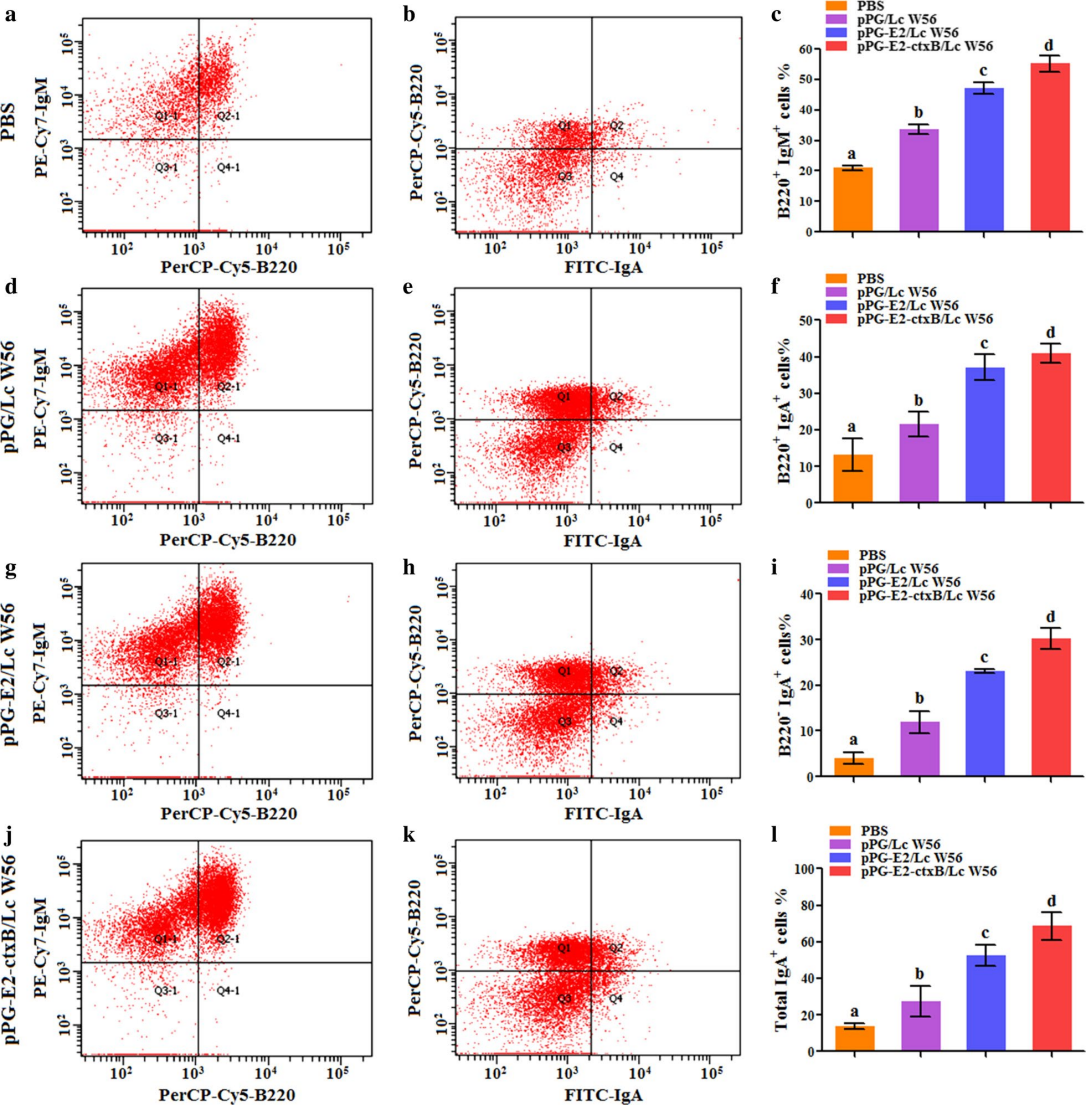
The percentages of B220^+^IgM^+^ B cells, B220^+^IgA^+^ B cells, B220^−^IgA^+^ plasma blast, and total IgA^+^ cells in the PPs of the mice in each group were determined on day 7 after oral vaccination. The letters (a vs. b, c vs. d) indicate significant difference (*p* < 0.05); the letters (a vs. c, b vs. c, b vs. d) indicate significant difference (*p* < 0.01); the letters (a vs. d) indicate significant difference (*p* < 0.001)

Firstly, we detected the level of T lymphocytes expressing Bcl-6 in the PPs of mice in each group using an IHC assay (Fig. 4a) and fluorescence IHC assay (Fig. 4b) on day 7 post-oral vaccination. The results showed that the amount of the Bcl-6-positive T lymphocytes was significantly increased in the PPs of the mice orally vaccinated with pPG-E2-ctxB/Lc W56 and pPG-E2/Lc W56, compared to that of the mice received pPG/Lc W56 and PBS, indicating that oral vaccination with the recombinant *Lactobacillus* pPG-E2-ctxB/Lc W56 and pPG-E2/Lc W56 can increase the expression of Bcl-6 in T lymphocytes in PPs. Secondly, we detected the level of CD4^+^CXCR5^+^ T cells in the PPs of mice in each group using flow cytometric analysis (Fig. 5a) on day 7 after oral vaccination. The results suggested that the level of CD4^+^CXCR5^+^ T cells in the PPs of the mice in the pPG-E2-ctxB/Lc W56 group was significantly higher than that of mice in the pPG-E2/Lc W56 group (*p* < 0.01) or pPG/Lc W56 group (*p* < 0.01) (Fig. 5b). This suggested that the expression of Bcl-6 promoted the differentiation of CD4^+^CXCR5^+^ T cells. Lastly, we detected the ability of B lymphocytes to proliferate and differentiate into IgA-secreting plasma cells in the PPs of mice in each group using flow cytometric analysis on day 7 after oral vaccination (Fig. 6). The results showed that the percentages of B220^+^IgM^+^ B cells (j and c), B220^+^IgA^+^ B cells (k and f), and B220^−^IgA^+^ plasma blast (i) in the PPs of the mice in the pPG-E2-ctxB/Lc W56 group were significantly higher than those of mice in the pPG-E2/Lc W56 group (g, h, c, f, and i) (*p* < 0.05), pPG/Lc W56 group (d, e, c, f, and i) (*p* < 0.01), and PBS group (a, b, c, f, and i) (*p* < 0.001). Moreover, the percentages of total IgA^+^ cells (l) in the pPG-E2-ctxB/Lc W56 group was significantly higher than in the pPG-E2/Lc W56 group (*p* < 0.05), pPG/Lc W56 group (*p* < 0.01), and PBS group (*p* < 0.001). Our data indicate that the recombinant *Lactobacillus* can effectively up-regulate the expression of Bcl-6, promote the differentiation of CD4^+^CXCR5^+^ T cells, enhance the proliferation ability of B lymphocytes, and drive their differentiation into IgA-secreting plasma cells.

### Determination of antigen-specific antibodies and cytokines induced by the recombinant *Lactobacillus* vaccine post oral vaccination

**Fig. 7:**
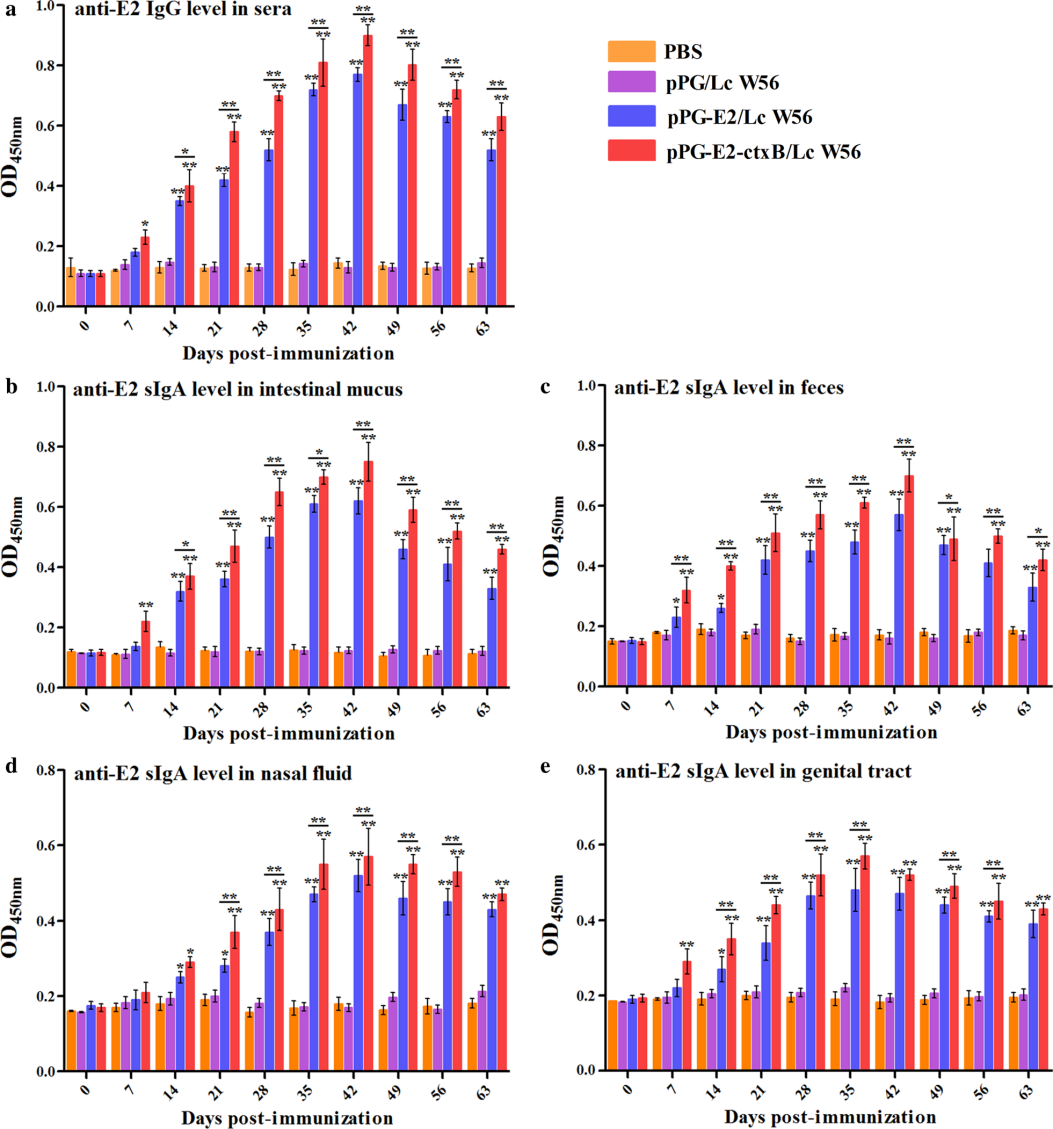
Determination of anti-E2 IgG antibody in sera (**a**) and anti-E2 mucosal sIgA antibody in intestinal mucus (**b**), feces (**c**), nasal fluid (**d**), and genital tract (**e**) of the mice in each group at different time points after vaccination by ELISA using BVDV as the coating antigen. Bars represent the mean  ±  SE of each group. *(*p* < 0.05), and **(*p* < 0.01)

**Fig. 8:**
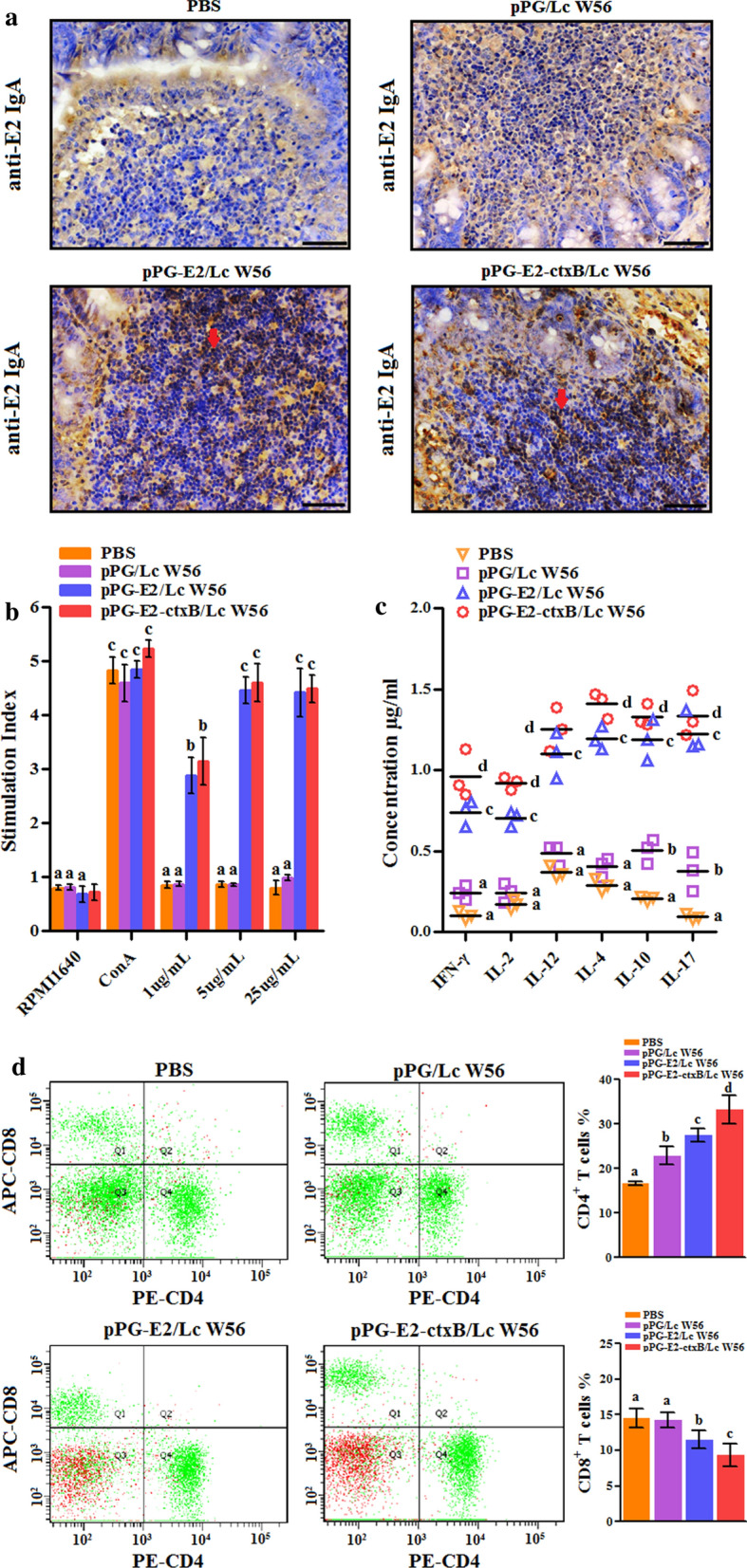
On day 42 post-primary vaccination: **a** antigen-specific IgA-secreting cells in the intestinal Peyer’s patches of the mice in each group detected by IHC assay; **b** the splenocytes obtained from the mice in each group stimulated by E2 protein, followed by lymphocyte proliferation determination by MTT assay; **c** the levels of cytokines IL-2, IL-4, IL-10, IL-12, IL-17, and IFN-γ in serum samples collected from the mice in each group determined by ELISA; **d** the percentage of CD4^+^ and CD8^+^ T cells in the spleen lymphocytes of the mice in each group determined by flow cytometric analysis (**d**). The letters (a vs. b, b vs. c, c vs. d) indicate significant differences (*p* < 0.05); the letters (a vs. c, b vs. d) indicate significant differences (*p* < 0.01); the letters (a vs. d) indicate significant differences (*p* < 0.001)

The levels of antigen-specific serum IgG and mucosal sIgA antibodies collected in each group at different days post-vaccination were detected by ELISA using BVDV strain ZD-2018 propagated in MDBK cells as coating antigen. Results showed that the antigen-specific IgG antibody in the pPG-E2-ctxB/Lc W56 group was significantly elicited on day 7 after oral vaccination (*p* < 0.05), compared to pPG-E2/Lc W56, Lc W56, and PBS groups. Furthermore, from the 14th days post the primary vaccination, significant levels of antigen-specific IgG antibody (*p* < 0.01) were detected in pPG-E2-ctxB/Lc W56 and pPG-E2/Lc W56 groups. In contrast, higher level (*p* < 0.01) of anti-E2 specific IgG antibody can be elicited by pPG-E2-ctxB/Lc W56 than pPG-E2/Lc W56 (Fig. 7a). Moreover, we found that significant levels (*p* < 0.01) of antigen-specific sIgA antibodies were detected in the intestinal mucus (Fig. 7b), feces (Fig. 7c), and genital tract (Fig. 7e) samples of mice orally vaccinated with the pPG-E2-ctxB/Lc W56 than that in mice from the pPG-E2/Lc W56, Lc W56, and PBS groups. Interestingly, significant levels (*p* < 0.01) of antigen-specific sIgA antibody were found to be elicited in the nasal mucosa by recombinant *Lactobacillus* vaccine (Fig. 7d). The immunogenicity of the pPG-E2-ctxB/Lc W56 inducing the production of antigen-specific sIgA antibody was greater than that of pPG-E2/Lc W56, and the level of antigen-specific IgG and sIgA antibodies observed in the Lc W56 group and PBS group has no significant changes before and after oral vaccination. Further, we detected the BVDV-neutralizing activities of serum IgG and mucosal sIgA antibodies harvested from different groups on the 42d post primary vaccination, and the BVDV-neutralizing dilution of IgG and sIgA antibodies collected from the pPG-E2-ctxB/Lc W56 and pPG-E2/Lc W56 groups were 1:128 ± 4.3 and 1:64 ± 2.9 (IgG), 1:96.2 ± 2.6 and 1:55.8 ± 3.1 (sIgA), respectively, while no neutralizing activity was detected in the Lc W56 or PBS groups. Moreover, compared with Lc W56 and PBS groups, significant levels of Th1-associated cytokines IL-2, IL-12, and IFN-γ, Th2-associated cytokines IL-4 and IL-10, and Th17-associated cytokine IL-17 were detected in the serum samples harvested from the mice in the pPG-E2-ctxB/Lc W56 group (*p* < 0.001) and pPG-E2/Lc W56 group (*p* < 0.01) (Fig. 8c). In addition, the level of cytokines stimulated by the pPG-E2-ctxB/Lc W56 was significantly higher (*p* < 0.05) than that stimulated by the pPG-E2/Lc W56. Further, we found that both pPG-E2-ctxB/Lc W56 and pPG-E2/Lc W56 were able to induce predominant Th2-type immunity (the ratio of IL-4/IFN-γ > 1). On the other hand, using the antigen-specific sIgA antibody obtained in this work, we examined the levels of anti-E2 sIgA-secreting cells in the PPs of the mice in each group using the IHC assay. The results suggested that the percentages of specific sIgA-secreting cells in the pPG-E2-ctxB/Lc W56 and pPG-E2/Lc W56 groups significantly increased, compared to Lc W56 and PBS groups (Fig. 8a). Overall, our results indicate that the pPG-E2-ctxB/Lc W56 can effectively induce antigen-specific mucosal and systemic immune responses using ctxB as adjuvant.

### Lymphocyte proliferation

The splenocytes obtained from the mice in different groups on the 42d post vaccination were stimulated using the E2 protein, followed by the determination of lymphocyte proliferation by MTT assay. As shown in Fig. 8b, the SI with different concentrations of the E2 protein in the pPG-E2-ctxB/LcW56 and pPG-E2/LcW56 groups was significantly higher (*p* < 0.05 or *p* < 0.01) than that in the Lc W56 and PBS groups. In addition, the percentage of CD4^+^ and CD8^+^ T lymphocyte subsets in the splenocytes of the mice in each group were determined by flow cytometry after oral vaccination (Fig. 8d). The data suggested that the percentage of CD4^+^ T cells in the pPG-E2-ctxB/Lc W56 was significantly higher than that in the pPG-E2/Lc W56 group (*p* < 0.05), Lc W56 group (*p* < 0.01), and PBS group (*p* < 0.001). However, the percentage of CD8^+^ T cells in the pPG-E2-ctxB/Lc W56 group was significantly lower than that in pPG-E2/Lc W56 group (*p* < 0.05), Lc W56 group, and PBS group (*p* < 0.01).

### Challenge experiment results

**Fig. 9:**
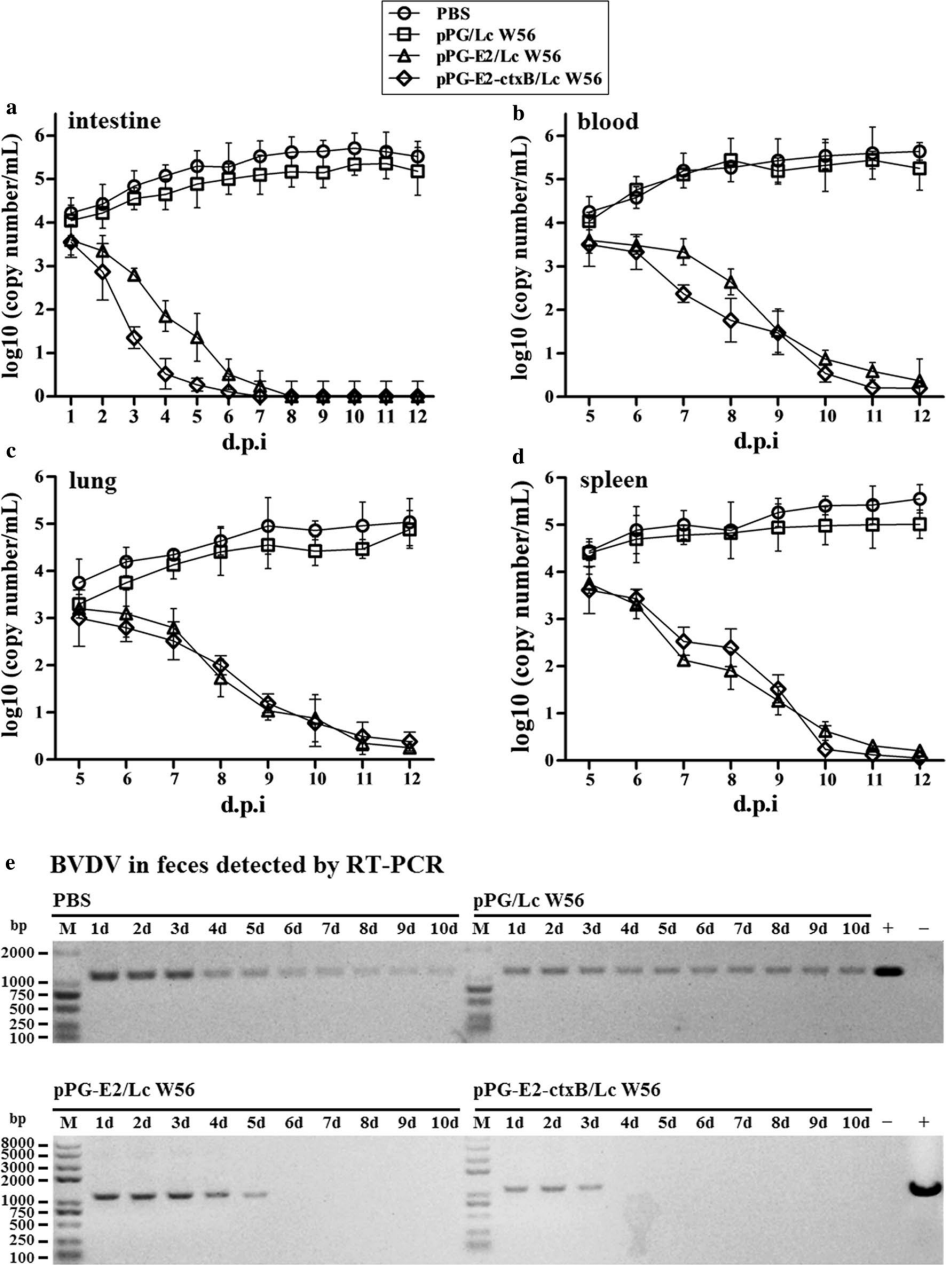
The virus loads in the intestine (**a**), blood (**b**), lung (**c**), and spleen (**d**) tissues of the mice in each group post-challenge with 10^5^ TCID_50_ BVDV, and the levels of virus excretion in feces of the mice in each group (**e**)

**Fig. 10:**
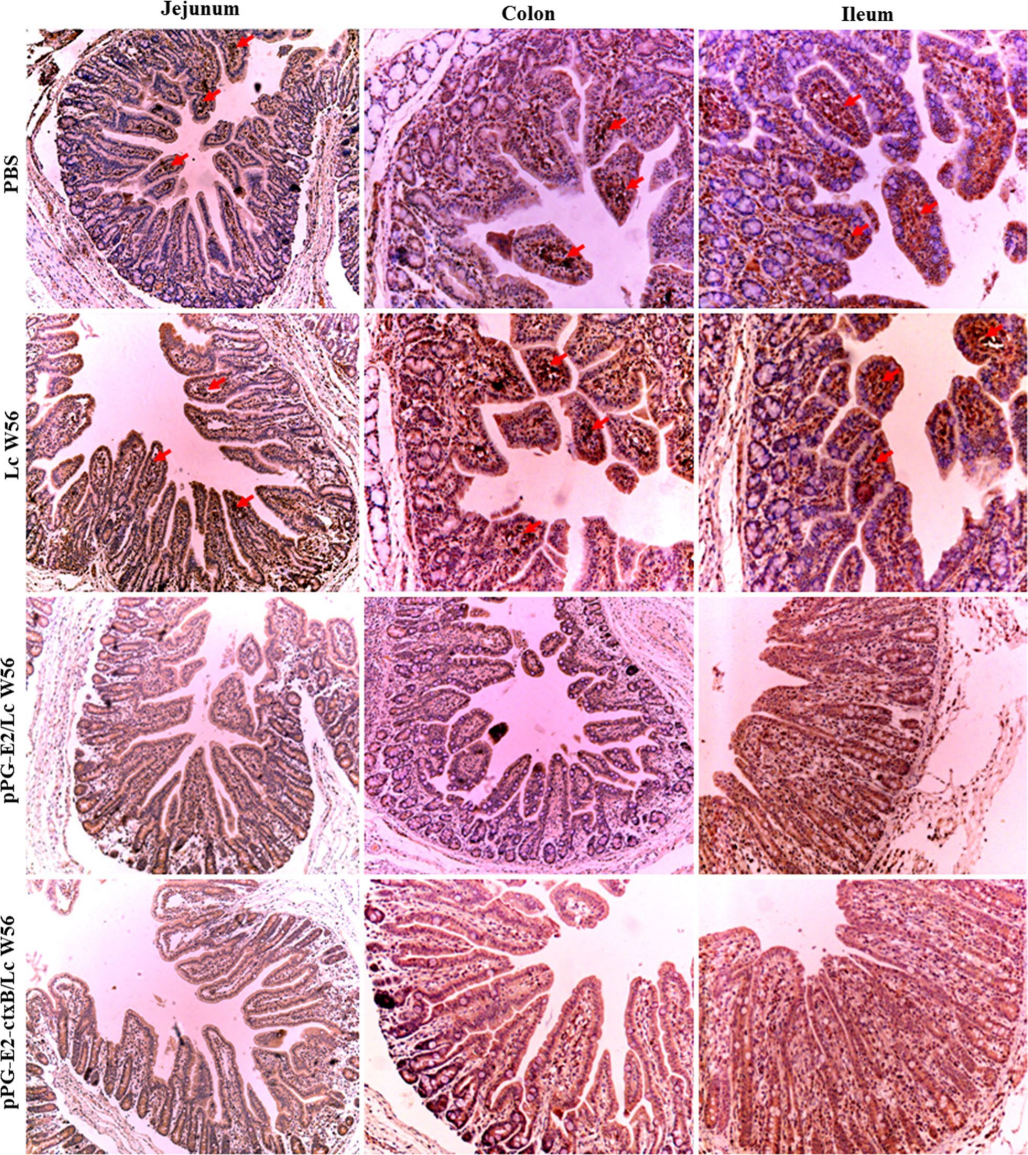
Immunohistochemistry assay used to detect the virus in the intestinal tract of the vaccinated mice in each group on day 12 post-challenge. No virus was detected in the jejunum, colon, and ileum of mice vaccinated with pPG-E2-ctxB/Lc W56 and pPG-E2/Lc W56, while large amounts of virus were observed in the mice in the PBS and Lc W56 groups

**Fig. 11:**
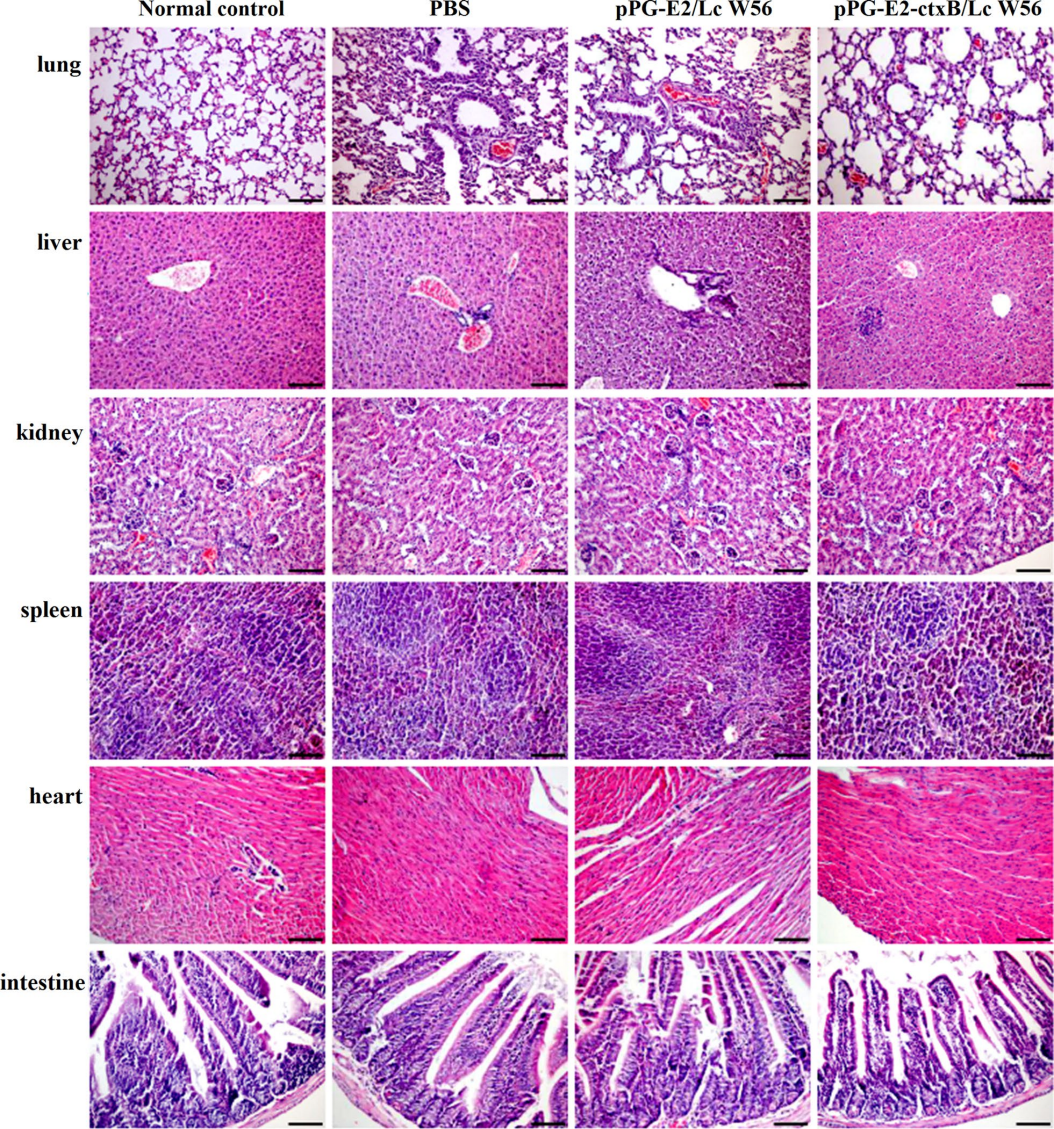
Histopathological changes in tissues of the vaccinated mice in each group post-challenge. There were no obvious histopathological changes observed in lung, liver, kidney, heart, spleen, and intestine tissues of the vaccinated mice in each group post-challenge with BVDV

We carried out a challenge experiment with 10^5^ TCID_50_ BVDV (200 µL per mouse) in different groups on the 42d post-oral vaccination. The viral loads in the intestine, blood, lung, and spleen of the mice were detected using RT-qPCR post challenge. Our results showed that in the pPG-E2-ctxB/Lc W56 and pPG-E2/Lc W56 groups, the viral loads gradually decreased in the intestine (Fig. 9a), blood (Fig. 9b), lung (Fig. 9c), and spleen (Fig. 9d) of the vaccinated mice, while the viral loads in either the Lc W56 or PBS groups increased gradually post-challenge. Notably, the most significant decline in viral loads was observed in the intestine. Moreover, we detected the virus excretion in the feces of the mice in each group using RT-PCR. As shown in Fig. 9e, the virus excretion in the feces samples collected from the mice orally vaccinated with pPG-E2-ctxB/Lc W56 and pPG-E2/Lc W56 was found to decrease gradually, while there were no viruses detected from day 4 in the pPG-E2-ctxB/Lc W56 group or day 6 in the pPG-E2/Lc W56 group onwards post-challenge, indicating efficient viral clearance. However, continuous virus excretion in the feces was observed in the Lc W56 and PBS groups. Furthermore, using the IHC assay, we detected the virus in the intestinal tract of the mice in each group on day 12 post-challenge. As a result, we found that no virus was detected in the jejunum, colon, and ileum of the mice vaccinated with pPG-E2-ctxB/Lc W56 and pPG-E2/Lc W56 orally, while large amounts of virus were observed in the mice of the PBS and Lc W56 groups (Fig. 10). Although mice can be used as an animal model for the evaluation of experimental BVDV infection, there were no obvious histopathological changes observed in the lung, liver, kidney, heart, spleen, and intestine tissues of the mice in each group after challenge with BVDV (Fig. 11).

## Discussion

To date, BVDV has caused significant economic losses to the cattle industry worldwide. Generally, the invasion of BVDV mainly initiates at mucosal surfaces of respiratory and intestinal tracts [13, 38]. Thus, the design of vaccines that can induce both a specific sIgA-based mucosal immune response and a specific IgG-based systemic immune response would be a promising and versatile strategy for the provision of effective immune protection against BVDV infection, preventing the virus from invading the body via the mucosa tissues and spreading to the systemic circulation. Although several modified-live virus and inactivated virus vaccines are currently commercially available and have contributed to the control of BVDV epidemic, these vaccines are mainly administered via intramuscular injection and generally fail to induce antiviral mucosal immunity [23]. Moreover, live-attenuated vaccines are associated with a high risk of viral virulence reversal, and are thus not completely safe for use in pregnant and PI cattle [39, 40]. Therefore, in this study, we constructed an oral recombinant *Lactobacillus* vaccine expressing BVDV E2 protein in conjunction with ctxB as an adjuvant and evaluated its immunogenicity for the induction of mucosal and systemic immune responses using mice as an animal model.

Currently, lactic acid bacteria are increasingly being recognized as promising candidates as antigen delivery carriers to express heterologous antigens in the field of vaccine development [41, 42], particularly *Lactobacillus* strains such as *Lactobacillus plantarum* [19, 43], and *Lactobacillus casei* [22, 23, 25, 36, 44]. Importantly, using lactic acid bacteria as live carriers not only allows for the delivery of antigens to induce specific immune response, but also plays a probiotic role in modulating non-specific immunity. In this study, the *Lactobacillus casei* strain W56 (Lc W56) was used as the antigen delivery carrier for the construction of a recombinant *Lactobacillus* vaccine, which was isolated from cattle feces and showed good probiotics, including good colonization ability in intestine, ability to promote animal growth performance, and protection animal against enteropathogenic bacteria infection. We systematically evaluated the tolerance of the genetically engineered *Lactobacillus* vaccine to several digestive environments, including a hypertonic environment, bile, and the intestinal fluid environment, particularly in the rumen fluid of cattle [23]. Our data demonstrated that the recombinant *Lactobacillus* vaccine had a good tolerance to digestive environments. Moreover, according to our results, the recombinant *Lactobacillus* vaccine was capable of entering the intestinal tract of the cattle via the rumen with an effective live bacteria number. Moreover, probiotics can aid in the digestion of food in the rumen of cattle, thus enhancing cattle health overall.

Using lactic acid bacteria to construct probiotic vaccine, the expression condition of target antigen is very important. Currently, xylose/nisin-based inducible expression systems are commonly used to construct probiotic vaccines [19, 43, 45]. However, prior to oral vaccination with these probiotic vaccines, antigen proteins must be subjected to being induced by some specific inducer, which has great limitations in the practical application of these probiotic vaccines [23]. In this study, the plasmid pPG-T7g10-PPT that was used to construct the recombinant *Lactobacillus* vaccine pPG-E2-ctxB/Lc W56 is a constitutive expression vector system constructed by our lab, which contains a T7g10 transcriptional enhancer, and an HCE constitutive strong promoter, thus, the target antigen could be constitutively expressed from the genetically engineered strain without the need for a specific inducer [20, 23, 25]. This provided a significant advantage in comparison to inducible expression systems. As powerful professional antigen-presenting cells in the intestine, dendritic cells (DCs) play an important role in regulating mucosal immune responses against infections by presenting antigens and promoting activation of T lymphocyte [46]. Therefore, we also evaluated the ability of pPG-E2-ctxB/Lc W56 to activate intestinal DCs. Our results demonstrated that the recombinant pPG-E2-ctxB/Lc W56 strain can effectively promote the maturation of intestinal DCs after oral vaccination, and the expression levels of CD40 and CD86 of intestinal DCs stimulated by the pPG-E2-ctxB/Lc W56 were significantly higher than that of other groups, indicating a good immunogenicity of BVDV E2 protein and effective immunoadjuvant activity of ctxB.

IgA is the predominant antibody at the mucosal surface and is produced locally at levels that exceed that of all of other immunoglobulins. So, as an effective oral vaccine, it will have to induce efficient mucosal IgA response. In this study, we detected the differentiation toward specific IgA-secreting cells in the Peyer’s patches stimulated by pPG-E2-ctxB/Lc W56. Bcl-6 is an important cellular factor, which is important for promoting differentiation of Tfh cells [47, 48]. Therefore, we first detected the changes of T lymphocytes expressing Bcl-6 in the PPs of the mice in these four groups after vaccination by IHC assay. Our results clearly demonstrated that a significantly increase in the amount of Bcl-6-positive T lymphocytes was observed in the PPs of mice vaccinated with pPG-E2-ctxB/Lc W56 orally. These results showed that vaccination with pPG-E2-ctxB/Lc W56 by oral route can up-regulate the expression of Bcl-6 in T lymphocytes, indicating that the Bcl-6 might play a role in the differentiation of CD4^+^CXCR5^+^ T cells observed. Subsequently, we evaluated the proliferation of B lymphocytes and their differentiation toward specific IgA-secreting plasma cells in the PPs of mice by flow cytometry analysis and IHC assay. Our results indicated significantly increased percentages of B220^+^IgM^+^ B cells, B220^+^IgA^+^ B cells, B220^−^IgA^+^ plasma blast, and antigen-specific sIgA-secreting cells in the pPG-E2-ctxB/Lc W56 group, demonstrating that the differentiation of CD4^+^CXCR5^+^ T cells stimulated by the pPG-E2-ctxB/Lc W56 enhances the proliferation of B lymphocytes and drives their differentiation into specific IgA-secreting plasma cells.

As an effective oral vaccine against BVDV infection, we expected our vaccine to induce both antigen-specific sIgA-based mucosal and IgG-based systemic immune responses. So, we determined the changes in the levels of antigen-specific sIgA antibody and IgG antibody in the samples collected from the mice in each group at different days post vaccination. We found that significant levels of mucosal sIgA antibody and serum IgG antibody were induced in the mice orally vaccinated with recombinant pPG-E2-ctxB/Lc W56 than other three groups, indicating a good immunogenicity of pPG-E2-ctxB/Lc W56 in conjunction with ctxB as an immunoadjuvant. Significantly, the antigen-specific mucosal sIgA antibody and serum IgG antibody induced by the recombinant *Lactobacillus* vaccine have BVDV-neutralizing activity in vitro, which is very important for the establishment of an effective vaccine against infections. This was significant for specific sIgA antibody elicited in the nasal mucosa. In addition, significant levels of Th1-associated cytokines, Th2-associated cytokines, and Th17-associated cytokine in the sera of the mice were induced by pPG-E2-ctxB/Lc W56, indicating that the recombinant *Lactobacillus* vaccine is capable of efficiently inducing cellular immune responses. Furthermore, we evaluated the immune protection effects induced by the recombinant *Lactobacillus* vaccine using mice as an animal model [49]. As a result, we observed that the viral loads in the intestine (feces), blood, lung, and spleen tissues of the mice in both the pPG-E2-ctxB/Lc W56 and pPG-E2/Lc W56 groups gradually decreased post-challenge. Notably, in the feces samples, no virus was detected from day 4 in the pPG-E2-ctxB/Lc W56 group and day 6 in the pPG-E2/Lc W56 group after BVDV infection, indicating efficient viral clearance. Significantly, on the 12d post-challenge, no virus can be detected in the intestine of mice vaccinated with pPG-E2-ctxB/Lc W56 and pPG-E2/Lc W56 by the IHC assay, while amounts of virus were observed in the Lc W56 and PBS groups.

## Conclusions

In conclusion, the recombinant *Lactobacillus* vaccine pPG-E2-ctxB/Lc W56 established in this work can effectively induce anti-BVDV mucosal and systemic immune responses and provide immune protection against BVDV infection in mice model by oral route, indicating a prospective strategy for vaccine development against BVDV infection. Nevertheless, the oral probiotic vaccination of cattle remains a serious challenge, although we are hoping to address this further in our studies that are currently under way.

## Data Availability

All the data analyzed have been included in this article.
