# Correction to: Immunogenicity evaluation of recombinant *Lactobacillus casei* W56 expressing bovine viral diarrhea virus E2 protein in conjunction with cholera toxin B subunit as an adjuvant

**DOI:** 10.1186/s12934-022-01928-9

**Published:** 2022-10-10

**Authors:** Shuo Jia, Xinning Huang, Hua Li, Dianzhong Zheng, Li Wang, Xinyuan Qiao, Yanping Jiang, Wen Cui, Lijie Tang, Yijing Li, Yigang Xu

**Affiliations:** 1 Heilongjiang Key Laboratory for Animal Disease Control and Pharmaceutical Development, College of Veterinary Medicine, Northeast Agricultural University, Harbin, People’s Republic of China; 2 Northeast Science Inspection Station, Key Laboratory of Animal Pathogen Biology of Ministry of Agriculture of China, Harbin, People’s Republic of China

## Correction to: Microb Cell Fact (2020) 19:186 10.1186/s12934-020-01449-3

Unfortunately, in the original publication [1] of the article, an error was found in Fig. 1b and Fig. 4b. In Fig. 1b, the background noises of images in panel anti-E2 and panel anti-ctxB were modified. Although the modification does not change the conclusions of the Western blotting experiment (anti-E2 and anti-ctxB), the authors had reperformed the experiment again and reassembled Fig. 1b with the new results. In Fig. 4b, the image of the panel pPG-E2-ctxB/Lc W 56 was one of the results of the pPG-E2/Lc W 56 group, which was mistaken by the students and should be corrected.

The corrected Figs. 1 and 4 are provided in this correction.

**Fig. 1:**
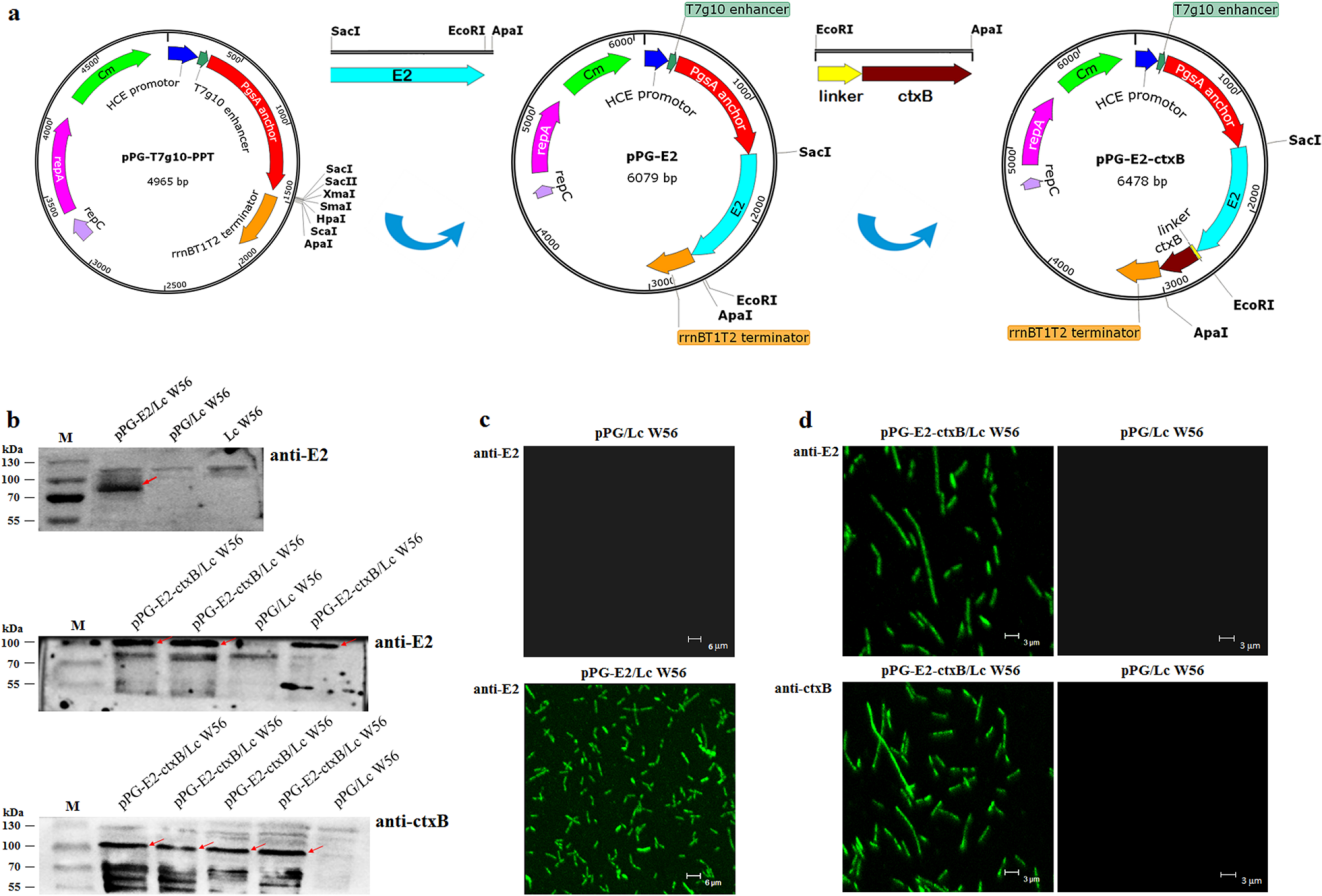
Construction of the recombinant *Lactobacillus* strains and identification of the proteins of interest expressed by the recombinant *Lactobacillus* strains. **a** Schematic illustration of the construction of the recombinant pPG-E2 and pPG-E2-ctxB plasmids. **b** Identification of the proteins of interest expressed by the recombinant strains pPG-E2/Lc W56 and pPG-E2-ctxB/Lc W56 using Western blot with mouse anti-E2 or anti-ctxB mAb, respectively. **c** Identification of the E2 protein expressed by pPG-E2/Lc W56 by IFA. **d** Identification of the fusion protein E2-ctxB expressed by pPG-E2-ctxB/Lc W56 by IFA

**Fig. 4:**
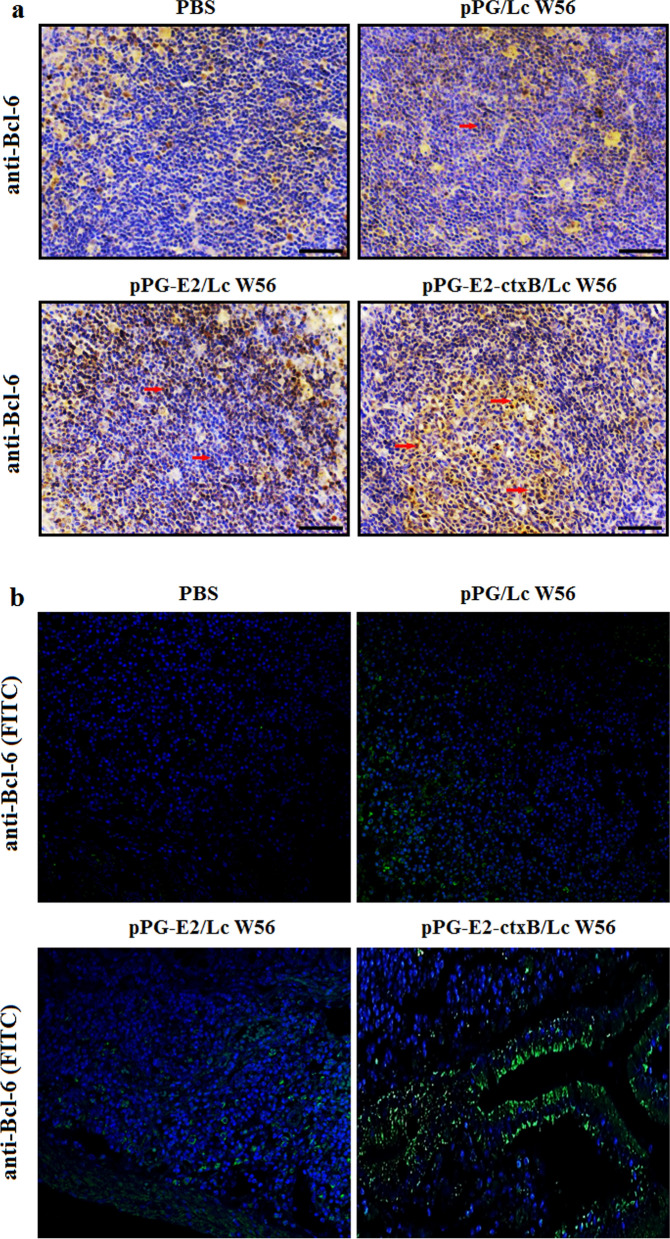
Detection of T lymphocytes expressing Bcl-6 in the PPs of the mice in each group on day 7 after oral vaccination by IHC assay (**a**) and fluorescence IHC assay (**b**)

## References

[1] Jia S, Huang X, Li H, Zheng D, Wang L, Qiao X, Jiang Y, Cui W, Tang L, Li Y, Xu Y (2020). Immunogenicity evaluation of recombinant *Lactobacillus casei* W56 expressing bovine viral diarrhea virus E2 protein in conjunction with cholera toxin B subunit as an adjuvant. Microb Cell Fact.

